# Static Progressive Orthoses for Elbow Contracture: A Systematic Review

**DOI:** 10.1155/2017/7498094

**Published:** 2017-09-07

**Authors:** Bin Chen, Jianhua Lin, Lifen Liu, Wenxin Niu

**Affiliations:** ^1^Department of Therapy, Shanghai Yangzhi Rehabilitation Hospital, Tongji University School of Medicine, Shanghai 201619, China; ^2^Tongji University School of Medicine, Shanghai 200092, China

## Abstract

**Background:**

As one of the most common musculoskeletal complications following trauma, elbow contracture is a frequent source of disabled daily activities. Conventional interventions are inadequate to provide favorable outcome. The static progressive orthoses are getting popular in the treatment of this problem.

**Objective:**

The purpose of this review was to assess the effectiveness of static progressive orthoses for elbow contracture.

**Methods:**

Literatures when written in English published during 1 January 1997 and 31 January 2017 were searched in the following databases: Web of Science, Cochrane Library, PubMed, and EBSCOhost. Articles are quality-assessed by two assessors, each article was summarized in evidence tables, and a narrative synthesis was also performed.

**Results:**

Ten clinical trials were included. The study design and outcome measures used varied. Significant immediate improvement in the range of motion was reported by all studies, and those effects were still significant at follow-up. No significant difference was shown between static progressive and dynamic orthoses for elbow contracture in one randomized control trial.

**Conclusions:**

Current low-quality evidence suggested that static progressive orthoses provided assistance for elbow contracture through improving range of motion. Further research is recommended using high-quality randomized controlled trials.

## 1. Introduction

As one of the most common musculoskeletal complications following trauma, elbow contracture is a frequent source of disabled daily activities [1, 2]. Due to the multitude of etiologies, the incidence of elbow contracture after trauma and surgery which requires surgical treatment is up to 12% [3]. Posttraumatic elbow contracture may result from both intrinsic and extrinsic factors. The intrinsic causes include intra-articular adhesions, articular malalignment, loss of articular cartilage, and a combination of the above, while the extrinsic causes contain capsular and ligamentous contracture, heterotopic ossification, extra-articular malunions, and soft-tissue contractures following burns [4]. Among those factors, capsular and soft-tissue contractures are considered the main causes of elbow contracture [5]. Recent reviews, however, suggested that most of elbow contractures are caused by a combination of both intrinsic and extrinsic factors [4]. More importantly, pain and swelling after trauma or surgery play an essential role in promoting the formation of contracture [2, 6].

The most direct consequence of contracture is the decreased range of motion (ROM). Normally, the range of elbow flexion 100° (30°~100°) and forearm rotation between 50° of pronation and 50° of supination is required for most daily activities, with a loss 50° flexion leading to an 80% loss of functionality [7]. Currently, there are two types of interventions for elbow contracture: operative and nonoperative treatment. The operative treatment involves open or arthroscopic release, arthroplasty, and manipulation under anesthesia [4]. Although these are efficient treatments, surgery and manipulation under anesthesia are complex and tend to cause neurovascular complications as well as recurrence [8]. The nonoperative treatment mainly involves passive or assistant movement, continuous passive movement, serial bracing, and static and dynamic orthoses [9, 10].

However, these interventions still have some limitations, such as time-consuming, therapist reliance, and lack of solid evidence support [11]. Also, the dynamic orthoses tend to cause soft-tissue injury and inflammation under a constant load to the joint, which results in low compliance [9, 12, 13]. Nowadays, static progressive orthoses have been used for the treatment of severe joint contracture after trauma and/or surgery. The underlying mechanism of static progressive orthoses on contracture is based on creep and stress relaxation [14]. As reported previously, it is faster to achieve the elongation with the stress relaxation principle [8, 15, 16].

According to Schultz-Johnson [17], static progressive orthoses have many advantages: (1) adjustable ROM and force, they could be adjusted to the maximum tolerable intensity to avoid pain and to have minimal damage; (2) controllable load, the patient could adjust load according to the subjective feeling; (3) higher tolerance and compliance; (4) mobility, the patient could do active exercise after removing the orthoses easily; and (5) effective, efficient, and economic, it requires less money and time by using static progressive orthoses.

Although many studies have reported that static progressive orthoses significantly improved the disabilities of patients with elbow contracture, there is still no consensus on the effectiveness and the protocols of static progressive orthoses [8, 16, 18]. Therefore, the purpose of this systematic review was to assess the effectiveness of static progressive orthoses on the management of patients with elbow contracture.

## 2. Methods

### 2.1. Search Strategy

A comprehensive literature search was conducted in the following bibliographic electronic databases: Web of Science, Cochrane Library, PubMed, and EBSCOhost. Articles were included when written in English, published between the beginning of 1 January 1997 and 31 January 2017. The following key terms and their combinations were used for literature search: static progressive/splint/splinting/brace/bracing/orthosis/orthoses, elbow, and range of motion. Two independent authors retrieved and selected the papers to determine the appropriateness based on the titles and abstracts. Then, full texts of these articles were assessed according to the inclusion and exclusion criteria. Any differences in the results of identification were resolved by consulting the third author.

The inclusion criteria were as follows: (1) subjects are patients with elbow contracture, (2) interventions include a static progressive technique, (3) outcome measure involves ROM, and (4) study design includes a clinical randomized control trial and pre-post intervention design.

The exclusion criteria were as follows: (1) case reports, comments, protocols, and review papers; (2) employment of invalid outcome measures; and (3) no static progressive technique as interventions.

### 2.2. Data Extraction and Assessment

Two independent reviewers extracted the data from the included studies by using the Population Intervention Comparison Outcome (PICO) method [19]. The data extracted from the studies included study design, sample size, population characteristics, intervention protocols, and outcome measures. Narrative and tabulation were used to conclude the effect of the static progressive orthoses. The included studies were qualitatively evaluated without attempting data synthesis.

All studies were assessed independently by two reviewers for methodological quality. The methodological index for nonrandomized studies (MINORS) was used to assess the articles without randomized design [20]. This appraisal tool consists of 8 items focused on stated aim, inclusion of patients, collection of data, outcome measure, unbiased assessment, follow-up, drop rate, and calculation of the study size. Each additional item is worth 2 points, for a maximum score of 16. Differences of the score were resolved by consensus between the two reviewers. The individual item scores and quality assessment score for each article are evaluated. Otherwise, level of evidence of the two controlled studies (randomized or nonrandomized controlled study) was used to assess the quality of the articles [21, 22]. The level of evidence ratings focused on patient enrollment, results, control group, study initiation, and outcomes. Higher levels of evidences could be more convincing to clinical practitioners [23].

## 3. Results

**Figure 1:**
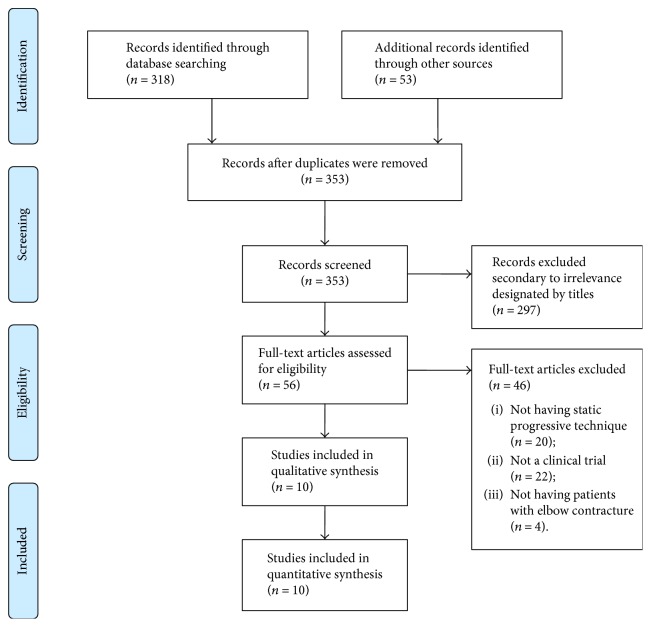
Flow chart of literature identification.

As shown in Figure 1, a total of 353 studies were identified from a comprehensive search. Firstly, the titles were read, and 297 articles were excluded for having no relevance to the primary objective of this review. Among the 56 trials left, 46 were excluded by reading abstracts and/or through full text. The reasons were displayed as follows: 22 studies were not clinical trials, 20 studies were excluded because they did not use the static progressive stretching in the method, and 4 studies were excluded for not treating elbow contracture. Thus, 10 clinical trials were included in this review (Table 1).

Of the ten studies included in this review, only two had controlled trials in the group [21, 22], whereas the remaining eight were of pre-post intervention design [9, 12, 15, 24–28]. The two controlled studies were scored as therapeutic evidence I and III, respectively [21, 22]. The individual item scores and total quality assessment score for the pre-post intervention study are summarized in Table 2. Only one study had a methodological score exceeding 60% [27], suggesting overall low methodological quality. All studies lost quality points for a lack of prospective collection of data. Only four studies explained the main outcome which should be in accordance with the question addressed by the study [12, 15, 27, 28]. Two studies did not report the inclusion and exclusion criteria [24, 28]. Assessors were not blinded in 7 studies [9, 12, 15, 24–26, 28], and four studies lacked appropriate follow-up [15, 24, 26, 27]. Another four studies were attributed to a high risk of bias in terms of high drop rate [9, 25, 26, 28]. Only two articles discussed their results on the basis of statistical significance, treating this equally to clinical relevance [25, 27].

In total, the included studies involved 289 patients (age: 8 ~ 77 years old), of which, 153 patients failed to improve by using standard physiotherapy [9, 12, 15, 21, 24, 27], whereas 89 patients received surgical release before static progressive stretching in other third studies [12, 22, 26].

Types of device used in the involved studies varied. There were four studies that investigated splint [21, 24, 25, 28] and two studies that examined the effects of orthoses [9, 26]. All other studies used mobilization brace [12], hinged external fixation [22], and Joint Active System [15, 27]. Most devices were custom-made based on the clients' elbow shape [9, 12, 21, 25, 26, 28]. In these studies, various intervention protocols were studied. For instance, some studies performed the treatment 30 mins/time, 3 times/day [15, 21, 25, 27], while the other studies applied the intervention between 6 hours and 20 hours per day [9, 12, 21, 24, 27]. The duration of the treatment was varied between 1 week and 9 months. Most of the patients insisted on the intervention at least 4 weeks [9, 12, 22, 24–27]. Five articles set the intensity to the most discomfort but without pain [9, 15, 24, 26, 27]. In addition, patients in most studies were instructed to do active or assisted active exercise during each session of treatment to maintain the stretching effects [9, 12, 22, 24, 26, 28].

A majority of studies have long-term follow-up after treatment [9, 12, 21, 22, 25, 28]. Significant immediate improvement in the ROM was reported in all studies, and those effects were still significant at follow-up. Two studies examined the satisfaction of interventions with significant results [15, 27]. In addition, two studies found a significant improvement in the upper limb function following the stretching intervention [21, 28]. The randomized control study found that both static progressive and dynamic orthoses could significantly improve posttraumatic elbow contracture, but the difference between the groups was not significant [21]. A slight increase in elbow motion in the group treated with static progressive orthoses than in the group not treated with static progressive orthoses was showed by another trial [22].

## 4. Discussion

This systematic review was aimed to appraise the effectiveness of static progressive orthoses on the elbow contracture. Despite the poor quality of included studies, this review suggested that static progressive orthoses could improve ROM for patients with elbow contracture. Although it is impossible to provide guidelines due to the diversity in therapy content and dosage, findings and suggestions are discussed in more detail within this discussion.

In these ten studies included, the subjects were reported to have experienced trauma, such as fracture and elbow dislocation. According to the reports, no study analyzed the outcome basing on the type or severity of the injury which could play a determinant role in the recovery process. If the injury occurred in the joint leading to the collapse of the joint surface, the prognosis could be limited when compared with that taking place in the ligament. A literature review concluded that orthoses could not provide an increase in joint motion due to intrinsic factors [29]. However, patients who had significant intrinsic factors did also have some degree of extrinsic factors that might respond to static progressive orthoses. This may suggest the demand of subgroup analysis to examine the specific effect on the dysfunction in different injured populations.

It is also noticed that the onset of the intervention varies among those studies. A few studies selected patients right after surgical treatment while other studies recruit patients who failed the 4-week to 11-week standard physiotherapy or had undergone elbow contracture between 52 days and 16.7 months. Although positive improvements have been reported in all these studies, for the studies that perform early intervention after surgery, the contribution of static progress orthoses to the improvement could be questionable as it is difficult to exclude the effects of other concurrent treatment.

As demonstrated in Table 1, the intervention protocols adopted in terms of the frequency, intensity, and duration of static progressive orthoses are discrepant among these studies. The treatment protocols were determined according to a physiotherapist's clinical experience or a manufacturers' suggestions, but the evidence was determined from the clinical study of high quality. Due to the discrepancy in intervention protocols, it is unable to synthesize the data to evaluate its effects on the elbow contracture. Muller et al. [8] suggested that the treatment should be 30 mins/time, about 3 times/day in each direction in consideration of the patient's compliance. Since there is no rigid randomized clinical trial to confirm this suggestion, its reasonableness remains unclear. However, a recent study found that higher stretch intensity or duration might be necessary to achieve better outcomes based on the creep and stress relaxation properties of the soft tissue [30].

All the studies included in this review used the ROM as the outcome measure, while two studies also employed functional scales and three studies assess the satisfaction with the treatment. It is well known that the clinical outcome should not only limit to impairment level as the importance of functional, psychosocial, and environmental aspects has been recognized in the International Classification of Functioning, Disability and Health (ICF) model. From current research, Disabilities of the Arm, Shoulder and Hand (DASH), American Shoulder and Elbow Surgeons evaluation (ASES-e), and Patient-Rated Elbow Evaluation (PREE) could enhance the ability of clinical practitioners and researchers to evaluate the patients with elbow pathology [31]. In order to comprehensively explore the effects of static progressive orthoses, these instruments meeting reliability and validity criteria should be recommended.

Out of the ten studies, five studies did long-term follow-up ranging from 6 months to 29 months and found that all the positive improvement remained at the follow-up [9, 12, 21, 25, 28]. It is interesting to notice that the duration of follow-up covers such a long time range. It is generally known that self-exercise could maintain the elbow function in the follow-up as ROM improves [29]. Compared to the long-term follow-up, it may be more valuable to investigate the short-term effect as the change of the length and flexibility of soft tissue will not take a long period if the patients kept doing appropriate range of motion exercise. Instead, the short-term measurement could reveal a more detailed time and range relationship of the elbow contracture which could provide a useful reference to optimize the treatment protocol.

Adverse events were mentioned in five studies [9, 12, 22, 26, 28]. The following complications are defined as adverse events in those studies: skin allergic reactions, scar breakdown, and nerve irritation. Only a few patients reported one of these complications during the treatment, but clinicians need to be aware of the risk of those complications and the measures to prevent or to deal with them when happened.

Another important finding was that all included articles were conducted between 2005 and 2012. It is somewhat surprising that no studies on static progressive orthoses for elbow contracture are performed in recent years. In contrast to our findings, however, a few studies confirmed the effectiveness of static progressive orthoses for shoulder and metacarpophalangeal joint contracture recently [14, 32]. This result may be explained by the fact that the effectiveness of static progressive orthoses for elbow contracture is generally confirmed.

Several methodological weaknesses may affect the strength of the evidence of these studies. Firstly, eight studies used pre-post intervention design without control groups, making it difficult to discriminate the real effect from the natural recovery or other factors. Secondly, in most of the studies, measurements were not conducted by independent assessors. Thirdly, the sample sizes of most of the studies in this review were not generally performed which could confound the efficacy of the program. As the drop rates were generally high, the clinical relevance of the effects often seems relatively small. Furthermore, there is a lack of a standardized treatment protocol of static progressive stretching, and it is impossible to synthesize the data for analysis.

This systematic review has the following strengths. Several databases were searched, and articles were selected independently. The methodological quality of the included pre- post intervention studies was assessed using the MINORS. It is considered that this article gives relevant additional information. More high-quality randomized control trials are required to increase the strength of evidence. The sample size should be calculated scientifically, and detailed intervention protocols are required for future studies. Future studies are also recommended to conduct a comprehensive outcome measurement with reasonable long-term follow-up to examine the effects of the static progressive orthoses for elbow contracture.

Finally, some factors may affect the outcomes of this review: (1) As the search strategy is limited to articles published in English, some studies may have been missed and (2) a meta-analysis is not appropriate due to study heterogeneity and low study quality.

## 5. Conclusion

To the author's knowledge, this is the first systematic review to consider the methodological quality for static progressive orthoses. Current low-quality evidence suggests that static progressive orthoses provided assistance for elbow contracture through improving ROM. Further research is recommended to examine the effectiveness of this treatment using high-quality randomized controlled trials.

## Figures and Tables

**Table 1:** Summary of evidence on splint for elbow contracture.

Reference	Study design	Subjects		Intervention			Evaluation		Results
		Number of subjects	Mean age	Type	Frequency	Duration	Outcome measures	Measurement schedule	
Ring et al. 2005 [22]	Nonrandomized control trail	Rx group: 23; C group: 19	39 (18–71)	Rx: hinged external fixation, active-assisted exercise; C: hinged external fixation (4–8 wks), active-assisted exercise; flexion and extension		Rx and C: 6 wks (4–8 wks)	ROM	Baseline, average of 39 mos	Between groups: ROM improvement with no significant difference (*p* > 0.05)
Parent-Weiss and King 2006 [26]	Pre-post intervention trial	28	41.2 (23–64)	Static progressive orthosis, supination and pronation	3-4 hs/time, removed 3 times for 1-2 hs each day; point of strong stretching sensation	12–24 wks	ROM (goniometer)	Baseline, end of Rx	Within group: supination and pronation ROM ↑
Doornberg et al. 2006 [25]	Pre-post intervention trial	29	38	Static progressive splinting, flexion and extension	30 mins/each direction, 3 times/day	4 mos (1–9 mos)	ROM (goniometer)	Baseline, average of 11 mos	Within group: ROM ↑ (*p* < 0.05)
McGrath et al. 2009 [27]	Pre-post intervention trial	38	44 (20–77)	Static progressive splinting, supination and pronation	30 mins/each direction/day; gradually progress to 3 sessions/day/each direction; pain-free, gentle stretch	12 wks (4–8 wks)	ROM; Likert scale (satisfaction)	Baseline, end of Rx	Within group: supination and pronation ROM ↑, the mean satisfaction score: 8.1 points
Bhat et al. 2010 [9]	Pre-post intervention trial	28	32 (8–68)	Turnbuckle orthosis, multiple directions; exercise	15 hs/daytime, removed 3 times for 1 h exercise; point of discomfort with no pain	5 mos	ROM (goniometer)	Baseline, end of Rx, average of 29 mos (follow-up)	Within group: flexion and extension group ROM ↑, rotation no effect
Ulrich et al. 2010 [15]	Pre-post intervention trial	37	45	Joint Active Systems (JAS); flexion and extension, when both required, larger deficit first 30 mins, then rest 15–30 mins, the other direction	1st wk, 1 session/day, 30 mins/session, increase the stretch to tolerance every 5 mins; 2nd wk, 2 sessions/day, then 3 sessions/day	10 wks (2–23 wks)	ROM (goniometer); 11-point ordinal Likert scale; analgesic use	Baseline, twice a week, until no ROM gains for 2 wks	Within group: ROM in flexion and extension ↑; 94% patients have satisfaction index scores; no patients require medication or an increase in the dosage
Marinelli et al. 2010 [12]	Pre-post intervention trial	42	Group A: ≥18	Groups A & B: mobilization brace; self-assisted movement; extension direction; group C: mobilization brace; self-assisted movement; CPM	Group A: night + removed 4 times/day for 1 h for exercise; after 4–6 wks, 4 times/day, 1 h/time + night; group B: same; group C: night + 5 times/day, 1 h/time, alternating flexion and extension	Group A: 3.5 mos; Group B: 3 mos; group C: 2 mos	ROM (goniometer)	Baseline, at least 6 mo follow-up	Within group: 3 groups ROM ↑
R. Suksathien and Y. Suksathien 2010 [24]	Pre-post intervention trial	3	17	Splint + motion exercise; extension direction	20 hs/day + night + every 1-2 hs was taken off for exercise daytime; adjust to the point of discomfort, but no pain	14 wks (11–20 wks)	ROM (goniometer)	Baseline, 14 wks (end of Rx)	Within group: ROM ↑
Liu et al. 2011 [28]	Pre-post intervention trial	14	41 (16–77)	Custom-made progressive stretching, flexion and extension; ROM exercise		7 days	ROM (goniometer); Mayo Elbow Performance score (MEPS)	Baseline, average of 14 mos (follow-up)	Within group: flexion and extension ROM ↑, mean MEPS score 92
Lindenhovius et al. 2012 [21]	Randomized control trial	Rx group: 35; C group: 31	≥18	Rx: static progressive splint; C: dynamic splint, multiple directions	3 times/day and 30 mins/time; 6–8 hs/day or night	Until no gains in AROM achieved in a thirty-day period	ROM (goniometer); Disabilities of the Arm, Shoulder and Hand (DASH)	Baseline,3 mos, 6 mos, 9 mos; 12 mos; baseline, 6 mos, 9 mos, 12 mos	Both ROM and DASH ↑; between groups: ROM at baseline, 3, 6, and 9 mos has no significant difference (*p* > 0.05); DASH at baseline and 12 mos has no significant difference (*p* > 0.05)

Rx: treatment; C: control; h(s): hour(s); wk(s): week(s); mo(s): month(s); BEO: before elbow orthosis treatment; DEO: during elbow orthosis treatment.

**Table 2:** Methodological quality ratings for each article.

Reference	Parent-Weiss and King 2006 [26]	Doornberg et al. 2006 [25]	McGrath et al. 2009 [27]	Bhat et al. 2010 [9]	Ulrich et al. 2010 [15]	Marinelli et al. 2010 [12]	R. Suksathien and Y. Suksathien 2010 [24]	Liu et al. 2011 [28]
A clearly stated aim	2	2	2	2	2	2	2	2
Inclusion of consecutive patients	2	2	2	2	2	2	0	0
Prospective collection of data	0	0	0	0	0	0	0	0
Endpoints appropriate to the aim of the study	0	0	2	0	2	1	0	2
Unbiased assessment of the study endpoint	0	0	2	0	0	0	0	0
Follow-up period appropriate to the aim of the study	0	2	0	2	0	2	0	2
Loss to follow-up less than 5%	0	0	2	0	2	2	2	0
Prospective calculation of the study size	0	2	2	0	0	0	0	0
Total score	4	8	12	6	8	9	4	6

MINORS (methodological index for nonrandomized studies) for 8 pre-post intervention design studies. The items are scored 0 (not reported), 1 (reported but inadequate), or 2 (reported and adequate).
